# Interdisciplinary risk counseling for hereditary breast and ovarian cancer: real-world data from a specialized center

**DOI:** 10.1007/s00404-022-06819-3

**Published:** 2022-10-28

**Authors:** Benedikt Zang, Malina Helms, Laura Besch, Nanette Kalmbach, Stephanie Stegen, Jens-Uwe Blohmer, Dorothee Speiser

**Affiliations:** 1 Charité-Universitätsmedizin Berlin, Zentrum Familiärer Brust Und Eierstockkrebs, Klinik Für Gynäkologie Mit Brustzentrum, Campus Charité Mitte, Charitéplatz 1, 10117 Berlin, Germany; 2 BRCA-Netzwerk E.V., Hilfe Bei Familiaeren Krebserkrankungen, Thomas-Mann-Str. 40, 53111 Bonn, Germany

**Keywords:** Hereditary breast cancer, Hereditary ovarian cancer, Genetic counseling, Digital tools

## Abstract

**Purpose:**

Hereditary breast and ovarian cancer has long been established to affect a considerable number of patients and their families. By identifying those at risk ideally before they have been diagnosed with breast and/or ovarian cancer, access to preventive measures, intensified screening and special therapeutic options can be obtained, and thus, prognosis can be altered beneficially. Therefore, a standardized screening and counseling process has been established in Germany under the aegis of the German Consortium for Hereditary Breast and Ovarian Cancer (GC-HBOC). As one of these specialized clinics, the HBOC-Center at Charité offers genetic counseling as well as genetic analysis based on the GC-HBOC standards. This analysis aims first at depicting this process from screening through counseling to genetic analysis as well as the patient collective and second at correlating the results of genetic analysis performed. Thus, real-world data from an HBOC-Center with a substantial patient collective and a high frequency of pathogenic variants in various genes shall be presented.

**Methods:**

The data of 2531 people having been counseled at the HBOC-Center at Charité in 2016 and 2017 were analyzed in terms of patient and family history as well as pathogenic variants detected during genetic analysis with the TruRisk^®^ gene panel when genetic analysis was conducted. This standardized analysis is compiled and regularly adjusted by the GC-HBOC. The following genes were included at time of research: *BRCA1, BRCA2, ATM, CDH1, CHEK2, PALB2, RAD51C, RAD51D, NBN,* and *TP53.*

**Results:**

Genetic analysis was conducted in 59.8% of all cases meeting the criteria for genetic analysis and 286 pathogenic variants were detected among 278 (30.3%) counselees tested using the TruRisk^®^ gene panel. These were primarily found in the genes *BRCA1* (44.8%) and *BRCA2* (28.3%) but also in *CHEK2* (12.2%), *ATM* (5.6%) and *PALB2* (3.5%). The highest prevalence of pathogenic variants was seen among the families with both ovarian and breast cancer (50.5%), followed by families with ovarian cancer only (43.2%) and families with breast cancer only (35.6%)—these differences are statistically significant (*p* < 0.001). Considering breast cancer subtypes, the highest rate of pathogenic variants was detected among patients with triple-negative breast cancer (40.7%) and among patients who had had been diagnosed with triple-negative breast cancer before the age of 40 (53.4%)—both observations proved to be statistically significant (*p* = 0.003 and *p* = 0.001).

**Conclusion:**

Genetic counseling and analysis provide the foundation in the prevention and therapy of hereditary breast and ovarian cancer. The rate of pathogenic variants detected is associated with family history as well as breast cancer subtype and age at diagnosis, and can reach considerable dimensions. Therefore, a standardized process of identification, genetic counseling and genetic analysis deems mandatory.

## What does this study add to the clinical work?

**Table:** 

The retrospective study depicts the process and challenges of genetic risk counseling for hereditary breast and ovarian cancer and emphasizes the relevance of such counseling using real world data. Adding to current knowledge it shows that prevalence of pathogenic variants is even higher than expected in certain breast cancer subtypes or family constellations.	

## Introduction

In recent years, the familial clustering of cancers, especially breast and ovarian cancers, has come more and more into view. Various factors have contributed to this heightened attention: The discovery of more and more relevant genes, but also through technical progress in genetic analysis (next-generation sequencing) and not least through the possibilities of targeted therapy, the topic of inheritance of tumor risks has arrived in daily clinical routine. With an estimated rate of 5–10% of 1.6 million breast cancer cases and 15–35% of 240.000 ovarian cancer cases worldwide, hereditary breast and ovarian cancer, diagnosed after detection of a pathogenic germline mutation in a relevant cancer susceptibility gene, constitutes a considerable number of carcinomas [1]. Since the discovery of the tumor suppressor genes *BRCA1* and *BRCA2* [2, 3], knowledge about these susceptibility genes has increased significantly. Much more insight has been gained on age dependence, histological subtype of carcinoma and immunohistochemistry [4, 5]. Furthermore, the list of susceptibility genes has been extended considerably in recent years, adding genes with more moderate penetrance such as *ATM*, *CDH1*, *CHEK2*, *RAD51C*, *RAD51D* [6, 7]*.* Correlations to other cancer entities like prostate cancer, pancreatic cancer, gastric and colorectal cancer as well as hereditary (cancer) syndromes like Lynch syndrome, Louis–Bar syndrome and Fanconi anemia have been observed [8–10]. Each of these genes has a specific profile including prevalence, associated carcinoma and/or syndrome and risk of disease which can be further categorized into moderate and high risk (17–30% and more than 30% lifetime risk) in the case of breast cancer [5, 7].

Consequently, the continued adjustment of preventive and prophylactic measures has been at the center of attention in specialized clinical care. Possible options are intensified screening and prophylactic operations. While early detection of ovarian cancer has not been proven reliable so far, prophylactic salpingo-oophorectomy remains the only effective option to significantly reduce the risk of ovarian cancer [11, 12]. Early detection of breast cancer equivalent to UICC Stadium 0 and I has been shown to be highly dependable in this collective of high-risk patients with a sensitivity of 84.5% [13], whereas efficacy of bilateral prophylactic mastectomy has only been proven for carriers of pathogenic variants (PV) in *BRCA1* [14, 15]. This last idea has been further corroborated by Heemskerk-Gerritsen et al. who demonstrated that bilateral prophylactic mastectomy only benefits carriers of PVs in *BRCA1*, while overall survival of PV carriers in *BRCA2* is not altered [16]. Taking into consideration the individual patient and family history in addition to the above-mentioned knowledge about susceptibility genes, risks involved and preventive measures, the counseling process as well as the decision-making process become increasingly complex for the physician and the counselee, respectively. On both sides, this can result in risk overestimation and unnecessary prophylactic operations [17].

With a view to this complex picture, the German Consortium for Hereditary Breast and Ovarian Cancer (GC-HBOC) has put great effort into establishing a standardized process for counseling, risk analysis, genetic analysis as well as preventive and prophylactic measures including a standardized screening process [7, 13]. The Charité center is part of the consortium and offers genetic counseling, genetic analysis and individualized preventive options according to the guidelines issued by the GC-HBOC.

The purpose of this paper is to depict the process of counseling at the HBOC-Center at Charité and to evaluate the results of this process in terms of the detection of PVs and associated factors on the basis of real-world data out of clinical care.

## Methods

**Fig. 1:**
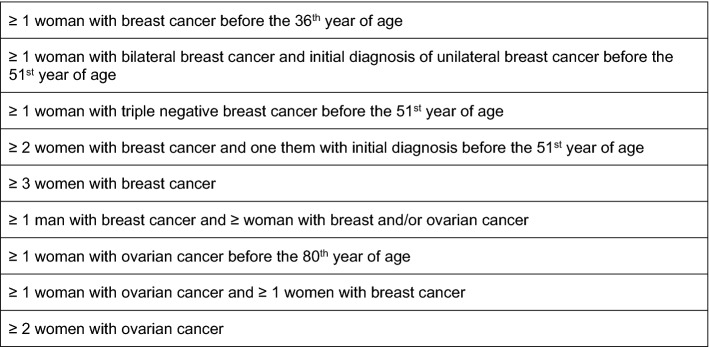
Inclusion criteria of the GC-HBOC at the time of genetic counseling and analysis conducted at the HBOC-Center at Charité in 2016 and 2017 (*GC-HBOC* German Consortium for hereditary breast and ovarian cancer). These criteria apply to all family members of genetic relation

The data of 2531 people, who received counseling in 2016 and 2017 at the HBOC-Center at Charité, were analyzed. Data on sociodemographics, family and patient history as well as details on breast cancer such as invasiveness and receptor status were gathered in a pseudonymised database. Breast cancer subgroups were composed incorporating invasiveness, receptor status and gender. Counselees who did not consent with anonymous data collection and analysis were excluded. For further statistical analysis, only counselees are complying with the inclusion criteria (IC) for genetic counseling by the GC-HBOC [18] or presenting with a familial tumor syndrome or having an attested PV in their family were considered. These inclusion criteria are based on the empirical observation of a heterozygosity risk of ≥ 10% in specific constellations of breast and/or ovarian cancer among family members of genetic relation (Fig. 1). Only one of the criteria (≥ 3 women with breast cancer) is included for historical reasons and does not meet the requirement of the mentioned heterozygosity risk [19, 20].

Genetic analysis was performed using the GC-HBOC’s TruRisk^®^ gene panel which is regularly adjusted to the latest state of research and included the following core genes at the time of research: *BRCA1, BRCA2, ATM, CDH1, CHEK2, PALB2, RAD51C, RAD51D, NBN* and *TP53* [7]*.* Detected genetic alterations are categorized based on the IARC-classification system for genetic variants established by Plon and colleagues [21]. Preferentially, genetic analysis of an available index patient, a family member who was diagnosed earliest in life and/or has the most severe form of breast or ovarian cancer and/or has the highest possibility of a PV in the family, was conducted. Should an index patient not have been available or willing to undergo genetic analysis, the family member of closest relation and with a corresponding heterozygosity risk was analyzed instead.

Statistical analysis was performed using IBM SPSS 25. Absolute and relative rates were computed for categorical variables, and mean, range and standard deviation were calculated for metric variables. To compare characteristics and PV detection rates among breast cancer subgroups, Chi-square-test was applied. Linear trend test was administered to examine for a correlation between age and mutation detection rate. Statistical significance was set at *p* < 0.05, respectively.

## Results

### Characteristics of counselees

2531 counselees were included in the analysis. The analysis of sociodemographic characteristics showed that counselees were predominantly female (*n* = 2493; 98.5%), had a mean age of 42.9 years (17–83 years; SD 12.2 years) and were for the most part employed (*n* = 1830; 72.3%). For 86.8% (*n* = 2198), it was the first consultation at the HBOC-Center. Of these, 85.3% (*n* = 1874) were the first in their respective families to receive counseling. Most counselees complied with the IC of the GC-HBOC (*n* = 2287; 86.8%). In almost all the families, involved cases of breast cancer (*n* = 2218; 97.0%) were reported. In a quarter of the families, additional cases of ovarian cancer (*n* = 613; 26.8%) were found. In a minor fraction, cases of ovarian cancer only were reported (*n* = 66; 2.9%).

### Rate of pathogenic variants detected

**Fig. 2:**
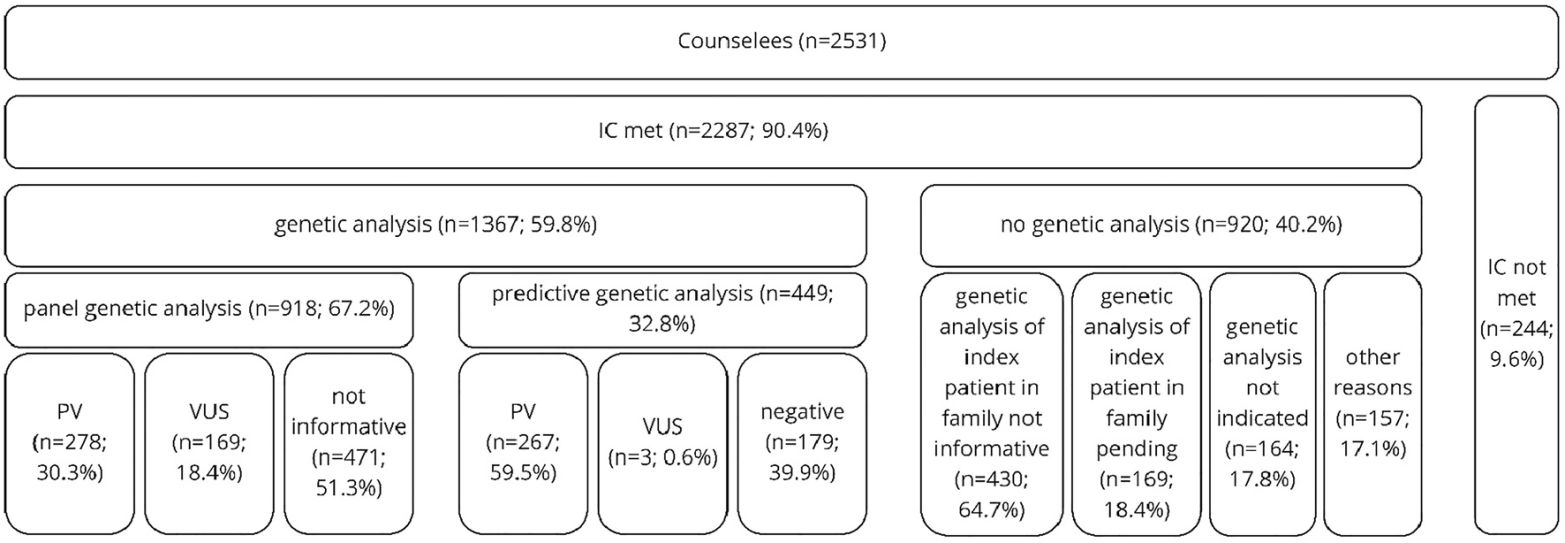
Genetic analysis conducted at the HBOC-Center at Charité in 2016 and 2017 (*IC* inclusion criteria of the German Consortium for hereditary breast and ovarian cancer, *PV* pathogenic variant, *VUS* variant of uncertain significance)

**Fig. 3:**
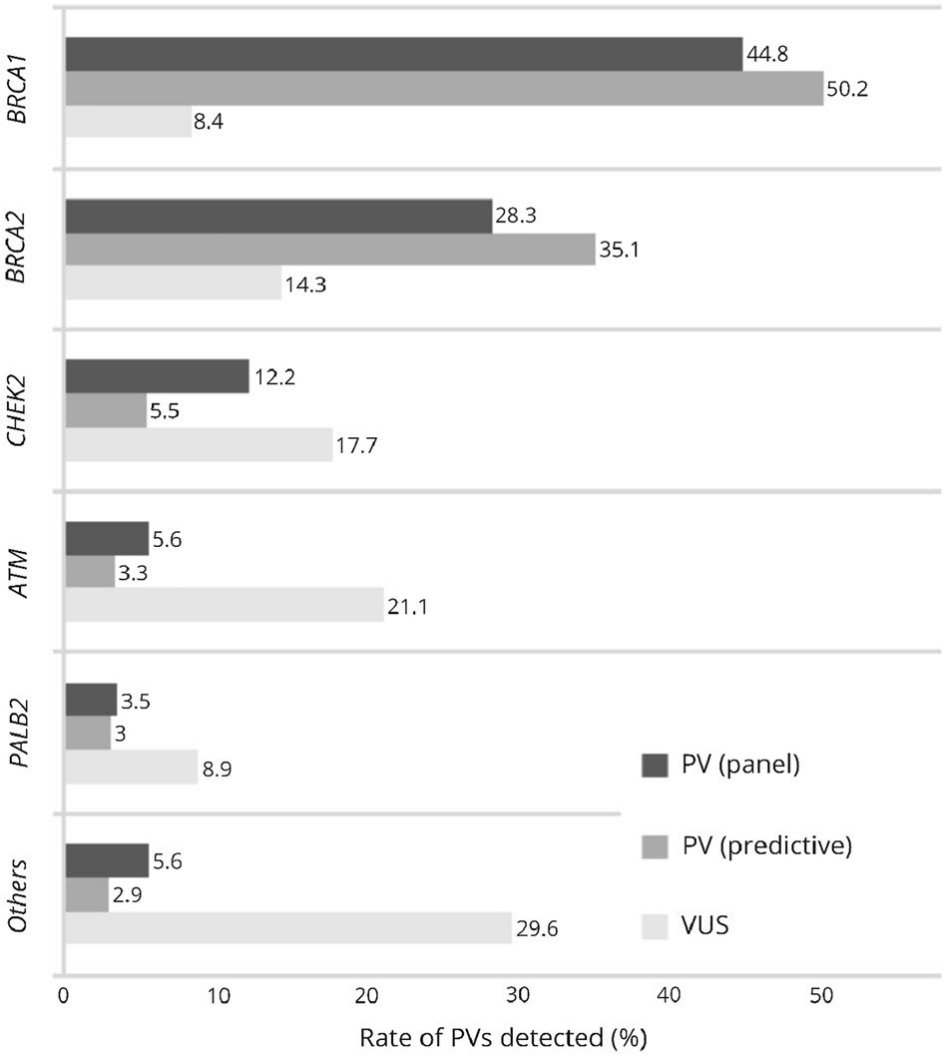
Variants detected at the HBOC-Center at Charité in the genes *BRCA1*, *BRCA2*, *CHEK2*, *ATM* and *PALB2* (*PV* pathogenic variant, *VUS* variant of uncertain significance)

Genetic analysis was conducted in 1367 (59.8%) cases of altogether 2287 counselees meeting the IC of the GC-HBOC. This includes 918 cases of complete panel analysis (67.2%) and 449 cases of predictive analysis (32.8%). Altogether, 286 PVs were detected among 278 (30.3%) counselees tested as index patients. Eight counselees (2.9%) presented with two PVs (Fig. 2). In decreasing order of prevalence, PVs were primarily found in the genes *BRCA1* (*n* = 128; 44.8%), *BRCA2* (*n* = 81; 28.3%), *CHEK2* (*n* = 35; 12.2%), *ATM* (*n* = 16; 5.6%) and *PALB2* (*n* = 10; 3.5%). Considering *BRCA1/2* alone, the frequency of PVs among counselees meeting the criteria for index analysis amounts to 22.8% (*n* = 209). 237 VUS were detected in 169 (18.4%) counselees tested as index patients. These were mostly found in the genes *ATM* (*n* = 50; 21.1%), *CHEK2* (*n* = 42; 17.7%), *BRCA2* (*n* = 34; 14.3%), *PALB2* (*n* = 21; 8.9%) and *BRCA1* (*n* = 20; 8.4%). Overall, 271 PVs were detected among 267 (59.9%) counselees through predictive genetic analysis, including four counselees with two PVs. Similar to the results of index analysis, the prevalence of PVs was most considerable in the genes *BRCA1* (*n* = 136; 50.2%), *BRCA2* (*n* = 95; 35.1%), *CHEK2* (*n* = 15; 5.5%), *ATM* (*n* = 9; 3.3%) and *PALB2* (*n* = 8; 3.0%) lastly (Fig. 3).

**Fig. 4:**
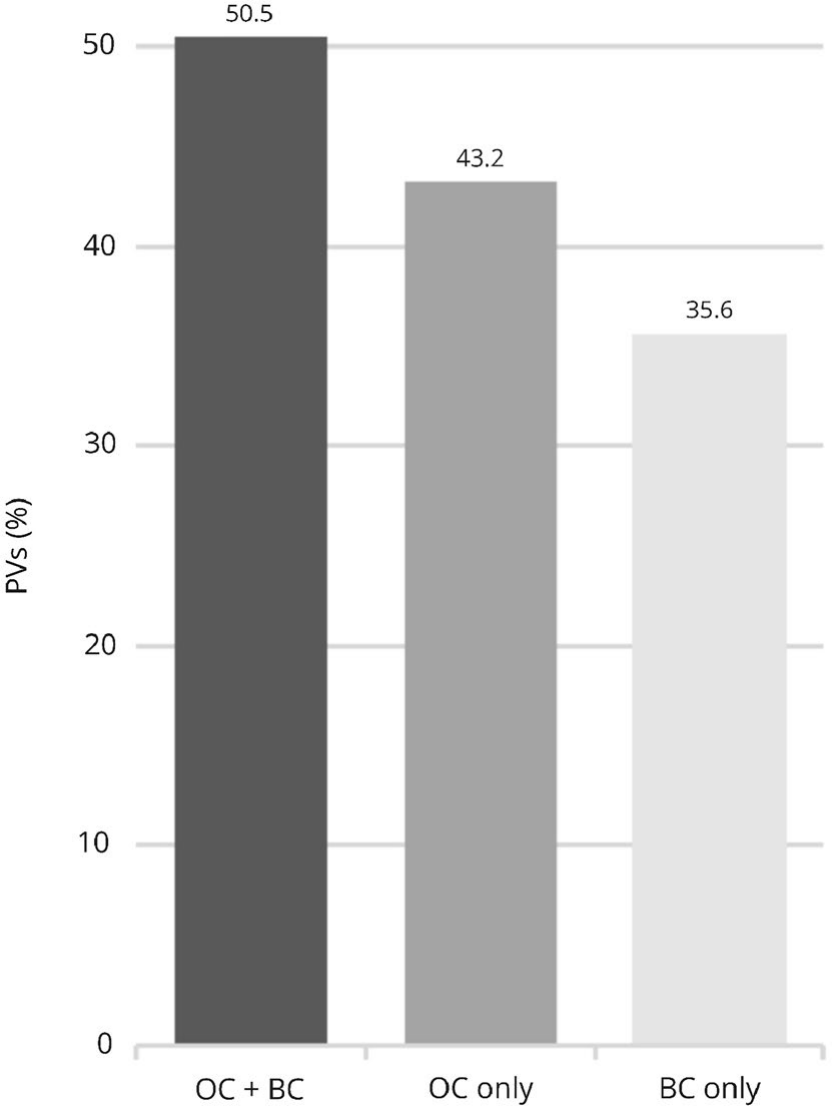
Pathogenic variants in analyzed families clustered based on occurrence of breast and ovarian cancer (*PV* pathogenic variants, *BC* breast cancer, *OC* ovarian cancer)

Correlating detection rates and constellation of cancer cases in the 1367 families tested, the highest prevalence of PVs with 50.5% (*n* = 187) was seen among the 370 families with both ovarian and breast cancer. Second, in 43.2% (*n* = 19) of the 44 families with ovarian cancer only and in 35.6% (*n* = 327) of the 919 families with breast cancer, only PVs were detected (Fig. 4). These differences associated with the respective entity of cancer in the families proved to be statistically significant (*p* < 0.001).

### Rate of pathogenic variants detected in breast cancer subgroups

Overall, 37.7% (*n* = 863) of counselees complying with the IC of the GC-HBOC (*n* = 2287, 86.8%) had been diagnosed with breast and/or ovarian cancer previously. Among these, there were 787 (91.2%) cases of breast cancer and 76 (8.8%) cases of ovarian cancer. On average, breast cancer was diagnosed at the age of 44.4 years (24–83 years) and ovarian cancer at the age of 50.8 years (17–75 years).

**Fig. 5:**
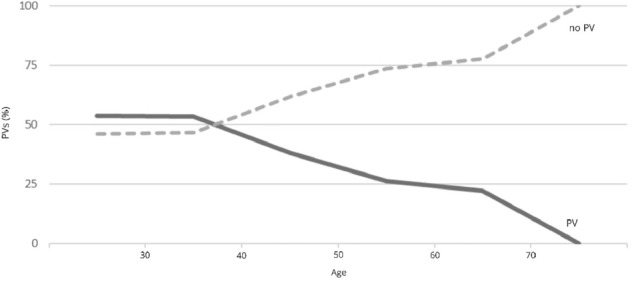
Rate of pathogenic variants among patients with triple-negative breast cancer depending on age (*PV* pathogenic variant)

95.7% (*n* = 753) of the counselees with breast cancer underwent genetic analysis. Subdividing those counselees according to receptor status, invasiveness and gender, the majority of 70.8% (*n* = 533) had hormone receptor positive and/or HER2-amplified breast cancer and a relevant part of 23.5% (*n* = 177) had triple-negative breast cancer, whereas ductal carcinoma in situ (*n* = 39; 5.2%) and male breast cancer (*n* = 4; 0.5%) were less common. Of those counselees with breast cancer who underwent genetic analysis (*n* = 753; 95.7%), the group with triple-negative breast cancer showed the most considerable rate of PVs detected (40.7%, *n* = 72). The other groups revealed lesser rates with 30.4% (*n* = 162) in the hormone receptor positive and/or HER2-amplified breast cancer group, 25.0% (*n* = 1) in the male breast cancer group and 12.8% (*n* = 5) in the ductal carcinoma in situ group. These differences were statistically significant (*p* = 0.003). Further analysis of the triple-negative breast cancer group revealed a relevant correlation between age of diagnosis and PV detection rate (*p* = 0.001). While 53.4% (*n* = 39) of counselees having been diagnosed with triple-negative breast cancer before the age of 40 had a PV, the rate of PVs detected decreased to 18.2% (*n* = 2) when the diagnosis of triple-negative breast cancer was made after the age of 60 (Fig. 5).

## Discussion

### Study population

The sample of counselees (*n* = 2531) included in this study is larger than the samples of other monocentric studies [22, 23]. Our sample size can partly be explained by the relatively large catchment area of our center which comprises Berlin and large parts of the adjacent federal states. Comparing the sociodemographic characteristics with other collectives of facilities of the GC-HBOC, the rate of female counselees of 98.5% (*n* = 2493), the mean age of 42.9 years and the employment rate of 72.3% (*n* = 1830) were relatively similar [20, 21]. However, with only 38 male counselees, a calculable analysis of male carriers of PVs was not possible despite the large study group. This underlines the well-established fact that men are much more reluctant to seek genetic advice than women [22]. Since male carriers of PVs have a higher risk for the development of breast cancer or other associated malignomas than the general male population and since their descendants (male or female) have a 50% chance of inheritance the PV, counseling more men with PVs ought to remain one of the primary objectives.

### Rate of pathogenic variants detected and therapeutic relevance

As previously demonstrated in several studies, PVs are most commonly found in the genes *BRCA1/2* [22, 23]. With a rate of 22.7% (*n* = 209) of PVs among index patients, our analysis confirms the considerably high prevalence of PVs in *BRCA1/2* in Germany as published by Meisel and colleagues as well as Kast and associates [24, 25]. Regarding PVs in *CHEK2* (*n* = 35; 12.2%) and *ATM* (*n* = 16; 5.6%), similar detection rates were ascertained by Schroeder et al., who analyzed test results of 620 counselees at two centers of the GC-HBOC [27]. Globally, however, there seems to be a substantial disparity as reported by Armstrong and colleagues, who conducted a systematic review to collate the prevalence of PVs in *BRCA1/2* [28]. Since they included studies with unselected study populations without familial breast and ovarian cancer, the prevalence rates are not comparable though.

By contrast, the present analysis lays emphasis on individuals that already have an increased risk for breast and ovarian cancer due to familial aggregation and meeting the GC-HBOC inclusion criteria. In a population properly counseled and selected by validated criteria, the frequency of a clinically relevant PV can amount to 30.3% (*n* = 278), as shown in this analysis, proving this process to be highly efficient. And taking into consideration triple-negative breast cancer alone, a rate of 40.7% (*n* = 72) for PVs detected indeed surpasses most previously reported data, yet a similar rate and an age dependence have been noted by Hahnen et al. in a comprehensive review [29]. Since triple-negative breast cancer before the 51st year of age alone is a sufficient inclusion criterion (Fig. 1), this observation emphasizes the relevance of this breast cancer subtype for genetic analysis. Whether an additional case of breast and/or ovarian cancer increases the probability of attesting a PV even further was not evaluated and poses need for more comprehensive investigation. However, with a mean age of 42.9 years and a PV detection rate of 53.4% (*n* = 39) among patients with triple-negative breast cancer before the age of 40, the present analysis supports the before mentioned age dependence. Furthermore, in light of the recently published data on the OlympiA trial [30] and the above-mentioned rate of 40.7% (*n* = 72) of PVs among counselees with triple-negative breast cancer as well as the predominance of the genes *BRCA1/2* among these PVs, the clinical relevance of genetic analysis as an essential part of therapy planning is underlined by these data. Since patients with germline PVs in *BRCA1/2* and residual invasive breast cancer or a CPS + EG score of 3 or higher after having received neoadjuvant chemotherapy clearly benefit from a 12 month adjuvant therapy with the PARP-inhibitor Olaparib, genetic analysis of all patients eligible for postneoadjuvant PARPi-therapy seems mandatory. Apart from the therapy with PARPi, the knowledge of an existing PV constitutes an essential factor when planning the operative therapy (i.e., prophylactic mastectomy and prophylactic salpingo-oophorectomy), as well as follow-up and screening (i.e., intensified breast cancer screening). The latter has been established to detect breast cancer reliably at an early stage [13] and can therefore benefit all carriers of a PV as well as their relatives afflicted by the same PV.

As reported by previous studies [31–33], the rate of PVs detected was highest among families with both ovarian and breast cancer (*n* = 187; 50.5%). Since, for instance, Kast et al. merely considered PVs in *BRCA1/2*, though the reported rate of PVs amounted to only 41.6%. Furthermore, the detection rate among families with breast cancer only was reported to range between 3.7 and 22.7% depending on additional factors like age at diagnosis and unilateral or bilateral occurrence [19]. While our study does not differentiate in the same fashion, it does include all ten core genes of the TruRisk^®^ panel relevant at the time of analysis. Altogether, 31.9% (*n* = 240) of counselees with breast cancer presenting at the HBOC-Center at Charité and meeting who underwent genetic analysis, were diagnosed with a clinically relevant PV. This substantially higher rate can be attributed to the population analyzed with a mean age of 42.9 years as well as the standardized implementation of the TruRisk^®^ panel comprising eight more relevant genes with partially considerable PV detection rates in addition to *BRCA1/2* [6]. The inverse correlation between age of diagnosis and rate of PVs detected proved to be statistically significant (*p* = 0.001), which concurs with other studies [34–36]. More interestingly, the detection rate among counselees between the age of 50 and 59 years in this subgroup came to 26.3% (*n* = 10) and 18.2% (*n* = 2) beyond the age of 60. Similar findings led to an expansion of inclusion criteria at the GC-HBOC for triple-negative breast cancer to be eligible for genetic analysis below the age of 60. Other authors arrived at similar yet slightly lower rates motivating them to propose an extension of genetic analysis for triple-negative breast cancer diagnosed before the age of 60 [34, 36], since the underlying heterozygosity risk of 10% for the indication for genetic analysis seems to be present in this subgroup. Therefore, the recommendation to offer genetic analysis to these patients has long been adopted by the National Comprehensive Cancer Network [36].

### Challenges and perspectives for the future

The assessment of PVs and especially of VUS underlies continuous scrutiny. Regarding the prevalence of VUS in *BRCA1/2* found in the present analysis (5.9%; *n* = 59), Meisel et al. found a slightly higher rate after evaluating the results of index analysis from 2000 to 2013 at one center of the GC-HBOC [26]. If only *BRCA1/2* are considered, the rate of VUS could be significantly reduced by reclassifications in the past [37]. The higher prevalence of VUS in the genes *ATM* (*n* = 50; 21.1%) and *CHEK2* (*n* = 42; 17.7%) can be attributed to the relatively common detection of alterations in these genes and the briefer period for which they have been analyzed standardly. Further and more extensive analysis such as the HerediVar project which aims at integrating bioinformatics and functional genomics into clinical classification of genetic variants promises to reduce the rate of VUS in the future [37]. For the time being however, only comprehensive counseling can help to apprehend the risk involved and thereby dissolve possibly unfounded fears.

The complexity of the counseling process is increasing due to a growing number of genes with clinical relevance as well as more differentiated prophylactic and therapeutic options. As a result of public coverage in recent years, the demand for genetic counseling seems to be increasing even more rapidly [37, 38]. Interestingly, a considerable number of counselees of the study population at hand (*n* = 244; 9.6%) did not meet the inclusion criteria according to the GC-HBOC but did wished to receive counseling due to a subjectively perceived risk. To accommodate the growing demand, the HBOC-Center at Charité has been offering video consultations since 2019. The use of such digital resources seems justified, given telephone consultation has been proven to be non-inferior [39–41]. To account for the complexity of the counseling process, the HBOC-Center has been developing and applying a digital counseling tool that is adjusted to individual needs and can be used permanently to promote self-efficacy of counselees.

## Conclusion

The present study has once more demonstrated the importance of interdisciplinary counseling as well as timely genetic analysis in the prevention and therapy of hereditary breast and ovarian cancer. As for the indication for counseling and analysis, more research is needed as well as the continued adjustment of inclusion criteria. To provide for the increasing need for counseling of healthy counselees as well as counselees after detection of pathogenic variants with clinical relevance, the HBOC-Center at Charité is putting great effort into developing digital tools to facilitate the time-consuming consultation process for doctors. The main focus of efforts to streamline the counseling process is to achieve a better understanding of risk among counselees and to reduce cancer worry, as well as to enable more targeted prevention measures based on a preference-sensitive decision-making process.
